# A new species of *Leucostethus* (Anura, Dendrobatidae) from Gorgona Island, Colombia

**DOI:** 10.3897/zookeys.1057.67621

**Published:** 2021-08-27

**Authors:** Taran Grant, Wilmar Bolívar-García

**Affiliations:** 1 Department of Zoology, Institute of Biosciences, University of São Paulo, 05508-090, São Paulo, SP, Brazil University of São Paulo São Paulo Brazil; 2 Grupo de Investigación en Ecología Animal, Departamento de Biología, Universidad del Valle, Calle 13 No. 100-00. Cali, Colombia. A.A. 25360, Cali, Colombia Universidad del Valle Cali Colombia

**Keywords:** Amphibia, Chocó, Colostethinae, *
Colostethus
*, Dendrobatoidea, *
Silverstoneia
*, taxonomy

## Abstract

We describe a new species of *Leucostethus* from Gorgona Island, a small (13 km^2^) island located 35 km from the Pacific coast of southern Colombia. The new species most resembles *L.argyrogaster* and *L.fugax* from western Amazonia at 340–870 m elev. in Peru and Ecuador, with which it shares pale ventral coloration and orange suffusion of the axilla, groin and concealed surfaces of the hind limb, but it is most closely related to *L.bilsa* from ca. 340 km SW in the southern Chocó at 420–515 m elev., northwestern Ecuador. We report miniscule white spots on the posteroventral surface of the thighs of the new species and, on the basis of our preliminary assessment of their taxonomic distribution, hypothesize that their presence is a synapomorphy of Dendrobatoidea with subsequent losses in a few groups. Given the apparent restriction of the new species to Gorgona Island, it is Vulnerable according to IUCN Red List criteria. In addition to naming the new species, we also propose the following new combinations: *L.alacris* (Rivero and Granados-Díaz, 1990) **comb. nov.**, *L.dysprosium* (Rivero and Serna, 2000) **comb. nov.**, and *L.yaguara* (Rivero and Serna, 1991) **comb. nov.**

## Introduction

Gorgona Island, located approximately 35 km from the Pacific coast of southern Colombia (Giraldo 2012), is a fragment of the Caribbean Large Igneous Province that formed in the Late Cretaceous and accreted to South America in the Eocene (Serrano et al. 2011). Although Gorgona is currently an island, the depths between it and the mainland are only ca. 60–120 m, so contiguous terrestrial habitat undoubtedly connected Gorgona and the mainland during the last glacial maximum (20–18 ka) and for a considerable time afterward (Fleming et al. 1998; Montealegre-Z et al. 2010). Indeed, Gorgona’s coral reef is estimated to date to only 2–3 ka (Glynn et al. 1982), placing a lower bound on Gorgona’s complete isolation from the mainland. Given its size (ca. 13 km^2^; Giraldo 2012), proximity to the mainland, and recent isolation, it is unsurprising that Gorgona’s biota is predominated by species that are widespread in the Chocó (Giraldo and Valencia 2012). Nevertheless, several putative endemics have been discovered on Gorgona, including species of Chelicerata (Lourenço and Flores 1989), Formicidae (Fernández and Guerrero 2008), Orthoptera (Montealegre-Z and Postiles 2010; Montealegre-Z et al. 2011; Baena-Bejarano and Heads 2015), and Psocodea (García Aldrete et al. 2011; Sarria-S et al. 2014; Manchola et al. 2014), although it is not uncommon for subsequent research to uncover species named from Gorgona on the mainland (e.g. Mendivil Nieto et al. 2017). Among vertebrates, the putative endemics include the catfish *Trichomycterusgorgona* Fernández & Schaefer, 2005, the anoles *Anolisgorgonae* Barbour, 1909 and *A.medemi* Ayala & Williams, 1988 (Phillips et al. 2019), the snake *Atractusmedusa*Passos et al., 2009, and an undescribed species of dendrobatid frog reported as “*Colostethus* sp. Gorgona” by Grant et al. (2017). The objective of the present paper is to formally name and describe this new species.

## Materials and methods

We conducted fieldwork on Gorgona Island (02°58'N, 78°10'W) from 23 to 28 May 2016. See Giraldo (2012) for a general description of the island and Vásquez-Vélez (2014) for a description of the vegetation. We euthanized specimens using 20% benzocaine (Orajel; Church & Dwight Co., Inc., Ewing, New Jersey, USA) applied to the skin or by pithing (McDiarmid 1994). Following preservation of one hind limb in 96–100% ethanol for DNA analysis and/or preservation of the skin in 100% methanol, specimens were fixed in formalin and subsequently transferred to 70% ethanol and deposited in the amphibian collection of the Colección de Prácticas Zoológicas, Universidad del Valle, Cali, Colombia (CPZ-UV). We also include in the type series one specimen from the National Museum of Natural History, Smithsonian Institution, Washington DC, USA (**USNM**).

We captured advertisement calls using a Tascam DR-40 linear pulse-code modulation (PCM) recorder and internal microphone at a sampling rate of 44.1 kHz with 16 bit encoding at 26.2°C ambient temperature. We also captured audio and video of the holotype vocalizing using a Canon EOS Rebel T3 and internal microphone and analyzed the audio (linear PCM recorder, 48 kHz sampling rate, 16 bit encoding). The calling males were observed directly and recorded at ca. 1 m distance. Acoustic analyses employed standard definitions and terminology for spectral and temporal variables (Köhler et al. 2017). We used Raven Pro 1.6 sound analysis software (Cornell Laboratory of Ornithology, NY) to score call duration (s), notes per call, note duration (ms), internote interval (ms), note repetition rate (notes/minute), number of pulses, fundamental and dominant frequencies (kHz), frequency modulation both within and among notes (kHz), and number and frequency of harmonics (kHz). We scored spectral parameters from spectrograms and power spectra (Hann window, 90% overlap, 512 point Fast Fourier Transformation resolution) and temporal parameters from expanded waveforms. The first 10–15 notes of each call were variable and differed from subsequent notes, all of which were emitted at higher amplitudes and varied little temporally and spectrally (i.e. they did not taper off toward the end of the call), so we report values for both entire calls and the first 10 and last 20 notes; as the latter values are most representative of the advertisement call, we used them for interspecific comparisons. We report statistical summaries as *x̄* ± SE.

Given that individual units of sound were consistently (i.e. both in the recorded calls and the many calls that were heard, but not recorded) emitted in bouts of < 30 s duration, we classified the individual units of sound as notes grouped into calls. This treatment is consistent with the interpretation by Vigle et al. (2020) of *Leucostethusbilsa*Vigle et al. 2020, which emitted units of sound in bouts of < 60 s. In contrast, other species of trans-Andean *Leucostethus* emit sounds at regular intervals in bouts varying from less than one minute to several minutes in duration, forming continuous series of variable length (a few to more than 300; Grant and Castro 1998); as such, in those species, Grant and Castro (1998) and Marin et al. (2018) considered calls to comprise a single note. For comparison, we treat the values reported by Grant and Castro (1998) and Marin et al. (2018) for “calls” as equivalent to the values reported by Vigle et al. (2020) and us as “notes.”

Information on phylogenetic relationships was taken from Grant et al. (2017), Marin et al. (2018), and Vigle et al. (2020). Although Grant et al. (2017) included the undescribed species from Gorgona Island in their *Colostethusfraterdanieli* group, Marin et al. (2018) subsequently transferred that group to *Leucostethus*, and we follow their taxonomy here. Character definitions follow Grant et al. (2006, 2017). For hand morphology, we follow Fabrezi and Alberch (1996) in considering finger I of other tetrapods to be absent in Anura and number fingers accordingly. As such, we follow Grant et al. (2017) in referring to the swollen third finger of earlier literature (e.g. Grant et al. 2006) as swollen finger IV. The webbing formulation is that of Savage and Heyer (1967), whereby webbing is quantified by the number and proportion of free phalanges (see also Myers and Duellman 1982; Savage and Heyer 1997). Jaw muscle terminology follows Haas (2001). We took the following measurements with digital calipers to 0.1 mm:

**SVL** snout–vent length

**FAL** forearm length from proximal edge of palmar tubercle to outer edge of flexed elbow

**HL** hand length from proximal edge of palmar tubercle to tip of finger IV

**TL** tibia length from outer edges of flexed knee to heel

**FL** foot length from proximal edge of outer metatarsal tubercle to tip of toe IV

**HW** head width between angle of jaws

**HLD** head length diagonally from corner of mouth to tip of snout

**EL** eye length from posterior to anterior corner

**END** eye-naris distance from anterior corner of eye to center of naris

**IND** internarial distance between centers of nares

**SL** snout length from anterior corner of eye to tip of snout

**IOD** interorbital distance

**TD** tympanum diameter

Unless otherwise noted, measurements and proportions are given only for adults and are summarized as *x̄* ± SE. Males with vocal slits on both sides of the mouth were scored as adults, those with only one vocal slit as sub-adults, and those lacking slits on both sides as juveniles. Females with expanded, convoluted oviducts and enlarged oocytes were considered to be adults, those with only weakly expanded, non- or weakly convoluted oviducts and poorly differentiated oocytes to be sub-adults, and those with small, undifferentiated oocytes and unexpanded, straight oviducts to be juveniles. Color in life is based on field notes and digital photographs.

We compare the new species to other species of *Leucostethus* sensu Marin et al. (2018) [viz. *L.argyrogaster* (Morales & Schulte, 1993); *L.bilsa*, *L.brachistriatus* (Rivero & Serna, 1986), *L.fraterdanieli* (Silverstone, 1971), *L.fugax* (Morales & Schulte, 1993), *L.jota*Marin et al., 2018, and *L.ramirezi* (Rivero & Serna, “1995” 2000)], to which we add *L.alacris* (Rivero & Granados-Díaz, 1990) comb. nov., *L.dysprosium* (Rivero & Serna, “1995” 2000) comb. nov., and *L.yaguara* (Rivero & Serna, 1991) comb. nov. Although the precise phylogenetic relationships of these three species have not yet been tested in quantitative phylogenetic analyses, Grant and Castro (1998) noted the resemblance of *L.alacris* and *L.yaguara* to *L.fraterdanieli* and Grant et al. (2017) considered the possibility that their Cordillera Occidental clade of *L.fraterdanieli* might correspond to one or both of those names. Similarly, *L.dysprosium* shares a complete, continuous, pale oblique lateral stripe with other species of *Leucostethus* (see Discussion) and appears to differ from other trans-Andean species of *Leucostethus* except *L.alacris* only in possessing more extensive toe webbing (see Rivero and Serna “1995” 2000: 49, fig. 2C). We also compare the new species to similar colostethines from the Chocó.

## Results

### Leucostethus siapida

Taxon classificationAnimaliaAnuraDendrobatidae

Grant & Bolívar-García sp. nov.

7857DA04-3F6E-5A7F-A73F-C498ACF66AC0

http://zoobank.org/757C0F3D-D62C-40E2-8F77-195C9234842D

Figs 1 , 2 , 3 , 4 , 5 , 6 , 7 , 8 , 9 , Tables 1 , 2 , 3

Colostethus sp. Gorgona: Grant et al. (2017)
Leucostethus sp. Gorgona: Marin et al. (2018), Vigle et al. (2020)

#### Type material.

***Holotype*.**CPZ-UV 7293 (field number WB 3045), an adult male collected by Taran Grant immediately west of the housing complex at El Poblado, Gorgona Island, Parque Nacional Natural Gorgona, Guapi, Cauca Department, Colombia, 02°58'00.6"N, 78°10'27.4"W, ca. 30 m elevation, 26 May 2016.

***Paratypes*.**CPZ-UV 5013–5014, CPZ-UV 7294–7297, collected at the type locality by Wilmar Bolívar-García, Taran Grant, David Andrés Velásquez-Trujillo, and Andrés Felipe Gómez Fernández, 26–27 May 2016. USNM 313893, collected by Humberto Granados Diaz, Gorgona Island, 1987.

#### Diagnosis.

A moderate-sized *Leucostethus* (maximum SVL: males 23.0 mm, females 25.8 mm) with complete pale oblique and ventrolateral stripes, dorsolateral stripe absent, finger IV of adult males very weakly swollen (barely discernible), throat of adult males bearing, at most, faintly stippled spotting or reticulation on throat, pale paracloacal spots present, axilla, groin, and concealed surfaces of hind limb suffused with orange, and toes II–IV with basal webbing.

#### Comparisons.

*Leucostethussiapida* sp. nov. differs from all congeners except the cis-Andean species *L.argyrogaster* and *L.fugax* in possessing orange axilla, groin, and concealed surfaces of hind limb (yellow in other species) and lacking conspicuous dark coloration on the throat of adult males (present in other species); it differs from both species in being larger (maximum SVL: *L.siapida* males 23.0 mm, females SVL 25.8 mm; *L.argyrogaster* males 19.8 mm, females 21.1 mm; *L.fugax* males 19.5 mm, females 20.1) and further differs from *L.fugax* in lacking conspicuous swelling of finger IV in adult males (strongly swollen in *L.fugax*).

Among the trans-Andean species of *Leucostethus*, *L.siapida* sp. nov. differs from all except *L.bilsa* (apparently unswollen) and *L.alacris* (unknown) in lacking conspicuous swelling of finger IV in adult (conspicuously swollen in *L.brachistriatus*, *L.dysprosium*, *L.fraterdanieli*, *L.fugax*, *L.jota*, *L.rodriguezi*, and *L.yaguara*). It differs from *L.bilsa* in the coloration of the axilla, groin, and concealed surfaces of the hind limb (orange in *L.siapida* sp. nov., “mustard-yellow” in *L.bilsa*; Vigle et al. 2020), definition and shape of the bright coloration of the axilla (suffused in *L.siapida*, discrete, forming well-defined crescent around dorsal, posterior, and ventral circumference of arm in *L.bilsa*), male throat coloration (at most faintly stippled spotting or reticulation on throat in *L.siapida* sp. nov.; more darkly spotted in *L.bilsa*), and female SVL (23.5–25.8 mm in *L.siapida*, 27.4–28.2 mm in *L.bilsa*; Vigle et al. 2020), as well as 6.25% of sites in the mitochondrial H-strand transcription unit 1 (146 of 2335 sites; Vigle et al. 2020). *Leucostethussiapida* sp. nov. differs from *L.alacris* in having only basal webbing between toes II and IV (moderate webbing between all toes, I 1–3 II 2–2.5 III 2.75–4 IV 4.5–3 V; Rivero and Granados-Diaz 1989).

Among species of *Leucostethus*, advertisement calls have been described for an apparently undescribed species from the Cordillera Occidental reported as *Colostethusfraterdanieli* (Grant and Castro 1998), *L.fraterdanieli* sensu stricto and *L.jota* (Marin et al. 2018), and *L.bilsa* (Vigle et al. 2020). The note repetition rate (*x̄* = 287.9 notes/minute) of *L.siapida* sp. nov. is more than twice that of the other species (*L.bilsa*: 81.2 notes/minute; *L.fraterdanieli* sensu stricto: *x̄* = 98.2 notes/minute; *L.jota*: *x̄* = 21.8 notes/minute; Cordillera Occidental species: 120.4–132.8 notes/minute). The advertisement call of *L.siapida* sp. nov. also has a higher pitched dominant frequency than all species except *L.jota* (peak frequency 4219–4737 Hz in *L.siapida* sp. nov., *x̄* = 4400 Hz in *L.jota*, < 3800 Hz in other species).

Among other anurans in the Chocó, *Leucostethussiapida* sp. nov. most resembles *Silverstoneiadalyi* and *S.nubicola* from the adjacent mainland, with which it shares dorsal and lateral coloration, orange suffusion of flash marks and limbs, a well-defined oblique lateral stripe, and a pale ventrolateral stripe (Grant and Myers 2013). It differs from these species in being larger (maximum SVL: *L.siapida* sp. nov., male 23.0 mm, female 25.8 mm; *S.dalyi*, male 17.9 mm, female 19.0 mm; *S.nubicola*, male 20.6 mm, female 21.9 mm) and lacking a conspicuously swollen finger IV (conspicuously swollen in both species). It further differs from *S.dalyi* in lacking a discrete, dark brown postrictal spot and from *S.nubicola* in bearing, at most, faintly stippled spotting or reticulation on the throat (male throat solid black, often extending posteriad into anterior belly in *S.nubicola*).

#### Measurements of holotype

**Figure 1.**
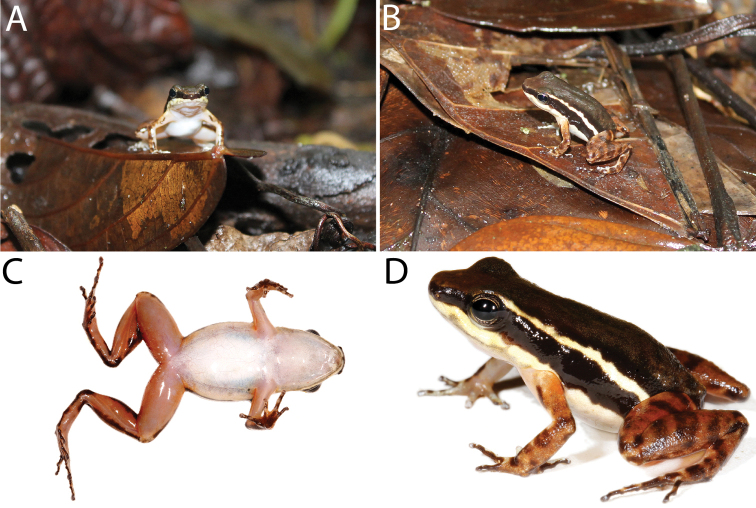
Holotype of *Leucostethussiapida* sp. nov. in life (adult male CPZ-UV 7293, 22.7 mm SVL) **A** frontal and **B** lateral views of holotype in situ while responding phonotaxically to playback of own vocalization **C** ventral view (note the suffusion of orange in axilla, groin, posteroventral thigh, and concealed surface of shank) **D** lateral view.

**(in mm).**CPZ-UV 7293 is an adult male (Fig. 1) with open vocal slits and melanized testes, SVL 22.7; FAL 5.0; HL 5.5; TL 10.2; FL 9.3; HW 7.8; HL 7.5; EL 2.9; END 2.5; IND 3.3; SL 4.0; IOD 2.4; TD 1.2.

#### Morphology.

**Figure 2.**
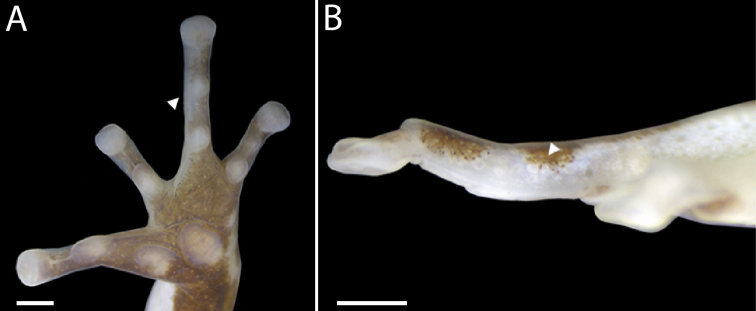
Right hand of *Leucostethussiapida* sp. nov. (CPZ-UV 7297, adult male) **A** hand in palmar (ventral) view (arrowhead indicates weak swelling) and **B** finger IV in pre-axial (medial) view (arrowhead indicates externally visible specialized mucous gland). Scale bars = 500 μm.

The following description is based on the five adult males and three adult females in the type series; measurements are reported in Table 1. Adult males 19.9–23.0 mm SVL (*n* = 5, *x̄* = 22.1 ± 0.56 mm); vocal slits present; swelling of finger IV barely discernible (Fig. 2); testis pigmentation variably present and absent (Table 2), forming dense brown or black reticulation when present (see also Remarks, below). Adult females 23.5–25.8 mm SVL (*n* = 3, *x̄* = 24.6 ± 0.67 mm), mature oocytes creamy yellow and pale brown, ca. 1.8 mm diameter; oviducts enlarged, convoluted, creamy white. Large intestine unpigmented.

**Table 1.** Measurements in mm (minimum-maximum, *x̄* ± SE) of the type series of *Leucostethussiapida* sp. nov. See text for measurement definitions.

	Males (n = 5)	Females (n = 3)
Snout-vent length	19.9–23.0, 22.1 ± 0.56	23.5–25.8, 24.6 ± 0.67
Forearm length	4.4–5.2, 5.0 ± 0.15	4.8–5.6, 5.2 ± 0.23
Hand length	4.7–5.5, 5.3 ± 0.15	5.5–5.8, 5.6 ± 0.09
Shank length	9.0–10.2, 9.9 ± 0.24	9.1–10.8, 10.1 ± 0.51
Foot length	8.1–9.5, 8.9 ± 0.24	8.4–9.8, 9.2 ± 0.41
Head width	7.1–7.8, 7.6 ± 0.13	7.5–8.7, 8.1 ± 0.35
Head length	6.5–7.5, 7.0 ± 0.19	7.0–8.4, 7.8 ± 0.41
Eye length	2.6–3.2, 2.9 ± 0.10	2.7–3.2, 3.0 ± 0.15
Eye-naris distance	2.0–2.5, 2.3 ± 0.09	1.9–2.7, 2.3 ± 0.23
Internarial distance	2.8–3.3, 3.1 ± 0.09	3.0–3.6, 3.4 ± 0.20
Snout length	3.4–4.0, 3.8 ± 0.12	3.2–4.5, 4.0 ± 0.39
Interorbital distance	2.3–2.6, 2.5 ± 0.05	2.4–2.7, 2.6 ± 0.09
Tympanum diameter	1.2–1.5, 1.3 ± 0.05	1.4–1.7, 1.6 ± 0.09

**Table 2.** Testis pigmentation in *Leucostethussiapida* sp. nov. See Remarks for comments on CPZ-UV 7295.

	Left	Right
CPZ-UV 5013	Pigmented	Unpigmented
CPZ-UV 5014	Unpigmented	Pigmented
CPZ-UV 7293 (holotype)	Pigmented	Pigmented
CPZ-UV 7295	Pigmented	–
CPZ-UV 7297	Unpigmented	Pigmented

Ventral and most dorsal surfaces smooth; exposed surface of shank with low, inconspicuous granules. Postrictal and cloacal tubercles absent.

Head width 33–36% of SVL and 1.0–1.2 times diagonal HL in males, 32–34% and 1.0–1.1 in females, IOD 29–36% of HW. Snout bluntly rounded in dorsal view, SL 46–58% of HL. Nares slightly flared, directed posterodorsad, EN 70–86% of EL and 57–66% of SL. Loreal region weakly concave, almost vertical. Canthus rostralis well defined, sharply rounded. Incomplete tympanic ring discernible externally along anteroventral half of tympanum. Eye length 38–48% of head length. Tympanum directed posterodorsad, TD 41–50% of EL in males, 52–53% in females. Supratympanic bulge associated with the underlying depressor musculature present. Teeth on maxillary arch present; median lingual process absent. Posterodorsal portion of tympanum concealed by m. depressor mandibulae fibers extending ventrad from origin on dorsal fasciae. Trigeminal nerve (V_3_) lateral to undivided m. levator mandibulae externus.

Hand length moderate, 22–25% of SVL and 1.0–1.1 times FAL. Relative appressed finger lengths IV > II > III = V (Fig. 2). Finger II 1.1 times longer than finger III (sensu Grant et al. 2006). Fingers III and V reaching distal half of distal subarticular tubercle of finger IV. All hand tubercles well defined and protuberant. Fingers II and III each with a single subarticular tubercle; fingers IV and V with two subarticular tubercles. Thenar tubercle elliptical, palmar tubercle elliptical or bluntly triangular. Fringes absent. Metacarpal fold absent. Discs weakly to moderately expanded, all bearing paired dorsal scutes. Carpal pad and black arm gland absent.

**Figure 3.**
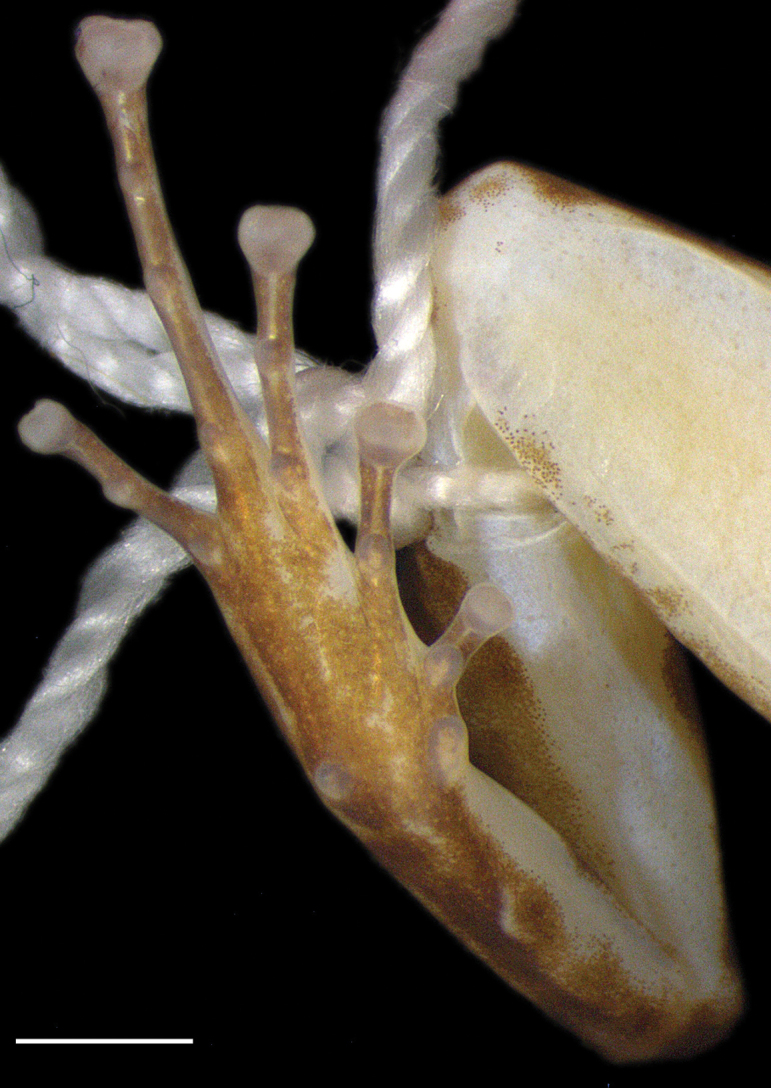
Right foot of *Leucostethussiapida* sp. nov. in life (adult male CPZ-UV 7293). Scale bar: 2 mm.

Tibia length 43–45% of SVL in males, 39–42% in females; FL 39–41% of SVL in males, 36–38% in females. Relative lengths of toes IV > III > V > II > I (Fig. 3). All foot tubercles well defined and protuberant. Toes I and II with one subarticular tubercle each, toes III and V with two, and toe IV with three tubercles. Webbing absent between toes I–II and IV–V; rudimentary webbing present between toes II–IV, giving formula II 2–3.5 III 3–4 IV. Fringes absent. Metatarsal fold absent. Tarsal keel well defined, short, strongly curved, tubercle-like, not extending from metatarsal tubercle, lying one-third of tarsal length from inner metatarsal tubercle. Discs bearing paired dorsal scutes; disc I weakly expanded, discs II, II, and V moderately expanded, disc IV greatly expanded. Distal thigh musculature with m. semitendinosus passing dorsad (ranid type), tendon of insertion bound to inner surface of mm. gracilis complex by secondary binding tendon.

#### Coloration in preservative.

**Figure 4.**
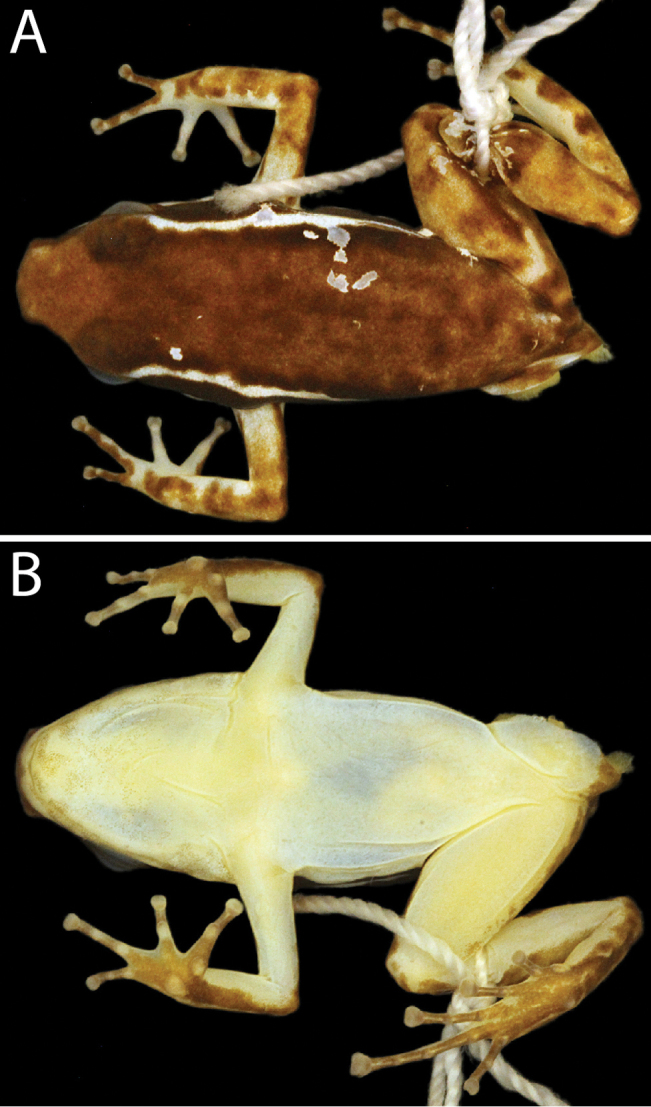
Preserved holotype (adult male CPZ-UV 7293, 22.7 mm SVL) of *Leucostethussiapida* sp. nov. in **A** dorsal and **B** ventral views.

Dorsal coloration brown with dark brown blotches (Fig. 4). Pale dorsolateral stripes absent. Eyelid dark brown, head and snout dorsally the same color as dorsum.

**Figure 5.**
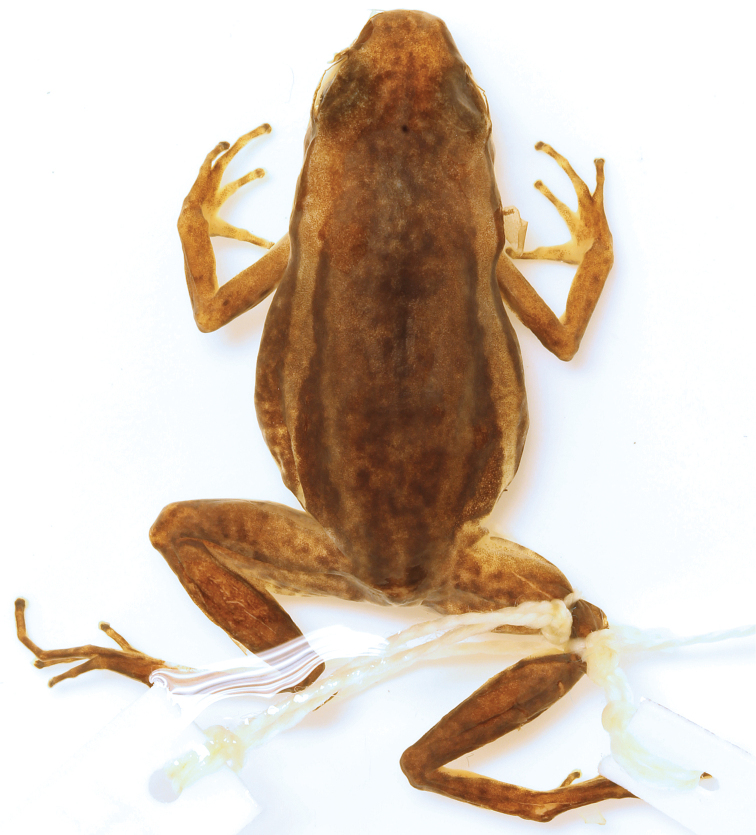
Dorsal view of *Leucostethussiapida* sp. nov. paratype USNM 313893 (adult female, 23.5 mm SVL).

Dorsal surface of thigh and shank and outer (exposed) surface of foot light brown with dark brown cross bands and blotches. Anterior surface of the thigh with prominent dark brown longitudinal stripe, delimited dorsally by narrow white line extending from groin and fading distally. Thigh ventrally immaculate. Most specimens with posterior thigh bearing elongate, tapered, pale sickle-shaped paracloacal stripe extending along proximal half to entire length of thigh, bordered dorsally by brown stripe extending along posterior shank and ventrally by solid or diffuse pale brown stripe; USNM 313839 lacking pale paracloacal marks, posterior thigh suffused with pale brown and diffuse dark brown blotches (Fig. 5). Ventral surface of thigh and concealed surfaces of shank and foot creamy white, free of melanophores. Plantar surfaces brown; contact surfaces of tubercles creamy white and gray. Webbing between toes III–IV creamy white, free of melanophores.

Dorsal surface of arm proximally pale, distally becoming pale brown with variably expressed dark-brown blotches to fingers IV and V, except USNM 313839 with entire dorsal surface of arm and fingers IV and V suffused with brown and irregular small dark brown spots. Anterior and posterior surfaces of upper arm with well-defined dark brown longitudinal stripes. Dark pigmentation on posterior surface extending distad to wrap around ventral and outer surface of elbow along posterior forearm and finger II. Anterior surfaces of forearm and dorsal surfaces of fingers II and III creamy white. Palmar surfaces pale brown; contact surfaces of tubercles creamy white.

Flank solid dark brown, divided diagonally by well-defined creamy white oblique lateral stripe from groin to posterior corner of orbit (i.e. not extending around canthus rostralis), except USNM 313839 with diffuse pale spotting below oblique lateral stripe and darker oblique lateral stripe. Oblique lateral stripe continuous, except left stripe of CPZ-UV 7294 with single narrow break at mid-length. Ventrolateral stripe (see Coloration in life, below) indistinguishable from immaculate venter (e.g. CPZ-UV 5014) or evidenced solely by sparse melanophores along ventral edge (e.g. CPZ-UV 7294), extending anteriad above arm insertion, below tympanum, and below eye to tip of snout, on head delimited ventrally by suffusion of sparse melanophores terminating beneath naris (i.e. upper lip at tip of snout creamy white, lacking melanophores). Dark brown of flank extending anteriad over tympanum to posterior edge of orbit and continuing over loreal region and around snout (encompassing nares) to form dark brown face mask.

Throat, chest, and belly creamy white, at most bearing faintly stippled spotting or reticulation on throat and along ventral edge of ventrolateral stripe.

#### Coloration in life.

**Figure 6.**
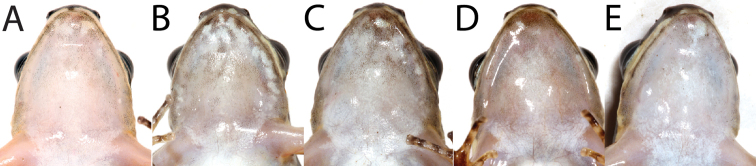
Variation in throat coloration of adult male *Leucostethussiapida* sp. nov. in life **A**CPZ-UV 7293 (holotype) **B**CPZ-UV 7295 **C**CPZ-UV 5013 **D**CPZ-UV 5014 **E**CPZ-UV 7297.

**Figure 7.**
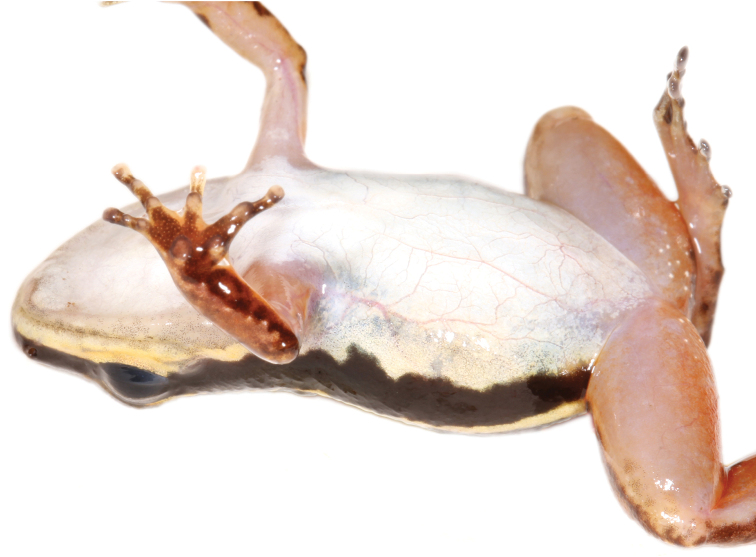
Ventrolateral view of *Leucostethussiapida* sp. nov. paratype CPZ-UV 7294 (25.8 mm SVL) in life showing difference between creamy white ventrolateral stripe and silvery white venter.

**Figure 8.**
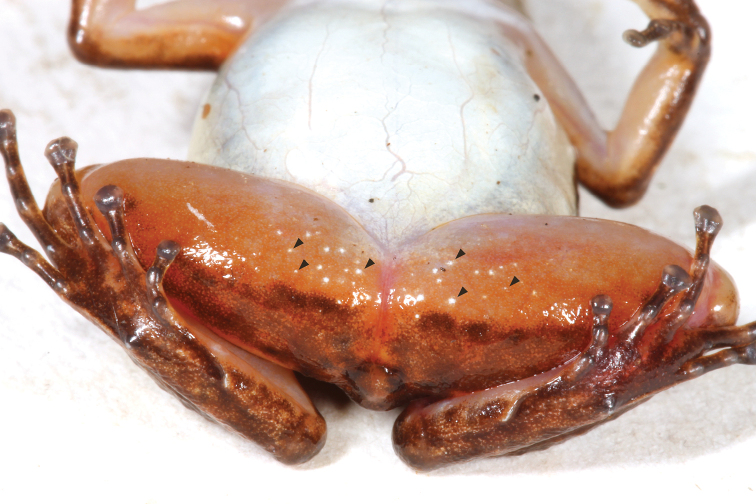
Posteroventral view of *Leucostethussiapida* sp. nov. thigh showing miniscule white spots (examples indicated by arrows; CPZ-UV 7296; 24.5 mm SVL).

Dorsum brown with blackish-brown blotches (Fig. 1). Dorsal surface of snout paler than dorsum, bearing diffuse creamy white spots suffused with brown. Flanks blackish-brown with creamy white to pale yellow oblique lateral stripe with faint suffusion of melanophores anteriorly (Fig. 1). Loreal region and tip of snout blackish-brown; area beneath tympanum, eye, and loreal region creamy white to pale yellow, delimited ventrally by diffuse suffusion of black. Venter silvery white. Throat with, at most, faintly stippled spotting or reticulation (Fig. 6). Ventrolateral stripe wavy, creamy white to pale yellow, differing from silvery white of venter, diffuse along ventral edge (Fig. 7). Dorsal surface of upper arm near insertion creamy white to pale yellow. Axilla, groin, posteroventral thigh, and concealed shank suffused with orange. Pale paracloacal marks conspicuous creamy white to inconspicuous pale brown. Ventral surfaces of thighs, ventral surface of upper arm and concealed surface of forearm translucent creamy pink, unpigmented. Posteroventral surface of proximal portion of thighs with few (< 10) to many (> 30) miniscule white spots, present in both sexes (Fig. 8). Iris pale gold with black speckles; pupil ring pale gold, complete.

#### Advertisement call.

**Figure 9.**
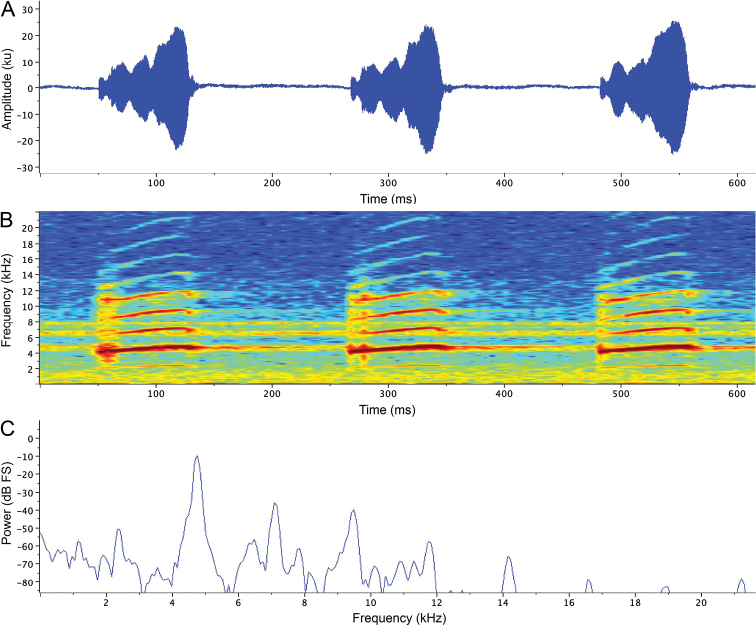
Advertisement call of *Leucostethussiapida* sp. nov. paratype CPZ-UV 7295 (19.9 mm SVL, 26.2°C ambient temperature; Suppl. material 2: Audio S2) **A** oscillogram and **B** audiospectrogram of three notes near the end of an 88-note call **C** power spectrum (spectrogram slice) at maximum amplitude of first note in **A** showing fundamental (2370 Hz) and dominant (4750 Hz) frequencies and harmonics (7120 Hz, 9490 Hz, 11820 Hz, 14190 Hz, 16610 Hz, 18970 Hz, and 21220 Hz). See text for analytical parameters.

On 26 May 2016, we recorded one complete call emitted by the holotype (CPZ-UV 7293; 22.7 mm SVL; Suppl. material 1: Audio S1, extracted from Suppl. material 4: Video S1) at ca. 11:30 h and two complete calls emitted by paratype CPZ-UV 7295 (19.9 mm SVL) at 15:45 h (call 1, Suppl. material 2: Audio S2) and 15:47 h (call 2, Suppl. material 3: Audio S3), respectively. Ambient temperature for all three calls was 26.2 °C. Notes are shown in Figure 9 and values for standard temporal and spectral variables are reported in Table 3. Notes are extensively amplitude-modulated throughout the three calls, resulting in highly irregular envelopes. Early notes are either pulsatile or comprise a single pulse, while subsequent notes become more structured, comprising 2–4 often irregularly shaped pulses. As expected, given their different body sizes, the fundamental and dominant frequencies are lower for the holotype (ca. 2160 Hz and 4300 Hz, respectively) than the paratype (ca. 2370 Hz and 4750 Hz, respectively); the note repetition rate of the holotype is also faster (last 20 notes emitted at 292.8 notes/minute by the holotype, 285.8 and 285.1 notes/minute by the paratype). Notes are frequency modulated, with the dominant frequency ascending approximately 150–200 Hz in the first 10 notes and 400–500 Hz in later notes (e.g. 3840 Hz to 4330 Hz in the holotype, 4210 Hz to 4670 Hz in CPZ-UV 7295; call 2, Suppl. material 3: Audio S3). In addition to the fundamental and dominant frequencies, 3–8 harmonics are evident in most notes.

**Table 3.** Characteristics of the advertisement call of *Leucostethussiapida* sp. nov. for the three recorded calls, including values for complete calls and the first 10 and last 20 notes, reported as minimum–maximum (*x̄* ± SE). Peak values are reported for the fundamental and dominant frequencies. For notes per call and note repetition rate *n* = 3 ; for variables related to individual notes, sample size is equal to the corresponding number of notes analyzed; internote internal sample size is three less than the corresponding number of notes. The fundamental frequency is reported only for the last 20 notes because some early notes were too weak for the fundamental frequency to be clearly identified.

Notes	Notes per call	Note repetition rate (notes/minute)	Note Duration (ms)	Internote interval (ms)	Fundamental frequency (Hz)	Dominant frequency (Hz)
All (*n* = 237)	73–88 (79.0 ± 4.6)	223.7–237.5 (232.7 ± 4.5)	10.9–98.9 (67.1 ± 1.4)	104.3–1308.3 (192.4 ± 9.2)	–	3220–4737 (4381 ± 20)
First 10	–	98.5–159.1 (133.9 ± 18.2)	10.9–67.9 (32.1 ± 2.1)	232.1–1308 (472 ± 45.3)	–	3563–4307 (4328 ± 59)
Last 20	–	285.1–292.8 (287.9 ± 2.5)	72.9–98.9 (87.0 ± 0.7)	106.6–140.3 (127.8 ± 0.9)	1969–2411 (2309 ± 15)	4219–4737 (4544 ± 24)

#### Etymology.

The specific epithet, *siapida* (*sia*, ‘arrow’^*^ or ‘wild cane’; *siapida*, ‘people of the arrow’ or ‘people of the wild cane’; Harms 1989; Barreña Agirrebeitia and Pérez-Caurel 2017), used as a noun in apposition, is a word in the Emberá Eperarã Siapidarã dialect and the name of the indigenous group located in the southern Chocó region of Colombia, specifically the modern Departments of Cauca, Nariño, and Valle del Cauca. The Siapida have visited Gorgona Island (*Thida* in Eperarã Siapidarã) for centuries or more and were almost certainly the first humans to encounter this species of frog.

#### Distribution and natural history.

**Figure 10.**
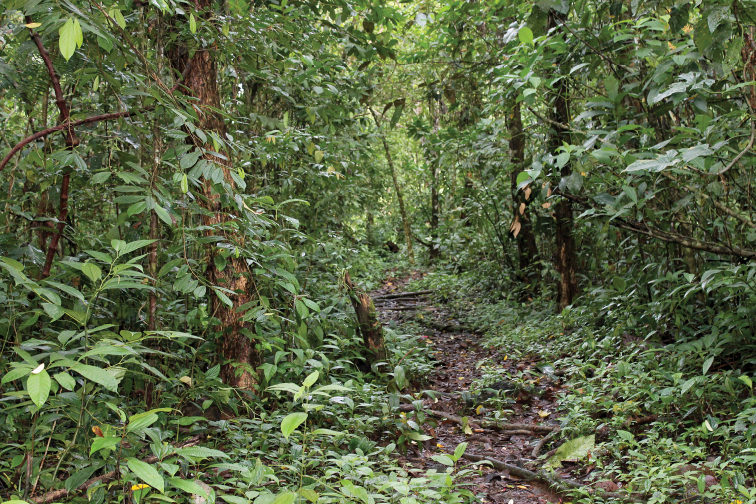
Habitat of *Leucostethussiapida* sp. nov.

Individuals were active during the day in and on leaf litter, low vegetation, and low objects on the forest floor (e.g. rocks, fallen sticks and logs, coconut shells; Figs 1, 10) independent from streams (i.e. >> 3 m from a stream). The holotype was filmed and photographed vocalizing while fully exposed on a large fallen leaf in a clearing (Suppl. material 4: Video S1), and other individuals were observed calling in the adjacent forest on leaf litter and low objects on the forest floor. The holotype responded to playback of his own vocalization both acoustically and phonotaxically, moving ca. 2 m toward the source of the vocalization to just a few centimeters from TG’s boots. Despite extensive searching and the abundance of *Epipedobatesboulengeri* on the previous days without rainfall, individuals of *Leucostethussiapida* sp. nov. were only observed after ca. 6 h of heavy rain between ca. 5:00 and 11:00 h on 26 May 2016.

#### Remarks.

The left testis of CPZ-UV 7295 is enlarged (1.4 mm diameter) and encapsulated by thick connective tissue beneath which the testis is pigmented, while the right testis appears to be absent. With the exception of their pigmentation, the testes of all other individuals are typical of this group (e.g. Marin et al. 2018; Vigle et al. 2020). Despite the testicular abnormality, CPZ-UV 7295 was observed and recorded vocalizing (see Fig. 9).

In life, the pale ventrolateral stripe is inconspicuous, but incontrovertibly present in all specimens. However, once pigmentation has faded in preservative, it is absent or inconspicuous in all specimens, with only a few scattered melanophores along its ventral edge remaining in some specimens to suggest a ventrolateral stripe once existed.

USNM 313839 differs in several respects from the other members of the type series and more closely resembles other trans-Andean species of *Leucostethus*, including the occurrence of diffuse pale spotting below the oblique lateral stripe and the oblique lateral stripe being extensively suffused with pale brown. Precise information on the area of the island where the specimen was collected is lacking, so it is unknown if the variation reflects highly localized geographic variation or widespread polychromatism. Nevertheless, the extent of variation that occurs among these specimens is well within that observed among conspecifics of other species (e.g. Vigle et al. 2020).

Gas-chromatography/mass spectrometry of the methanol extract of the skin of adult male paratype CPZ-UV 7297 failed to detect any alkaloids (R.A. Saporito, personal communication). Although this finding rules out the lipophilic alkaloid-based chemical defense found in many dendrobatids (e.g. Saporito et al. 2012), it is possible that tetrodotoxin or some other hydrophilic compound might be present, as reported for some species of *Colostethus* (Daly et al. 1994; Grant 2007).

## Discussion

Although phylogenetic analyses strongly place *Leucostethussiapida* sp. nov. as part of the trans-Andean *Leucostethusfraterdanieli* group (the *Colostethusfraterdanieli* group of Grant et al. 2017; i.e. all species, except *L.argyrogaster* and *L.fugax*), its precise phylogenetic relationships are unclear. Grant et al. (2017) found it to be the lowland sister of the remainder of the otherwise montane trans-Andean *Leucostethus*, whose species occur from ca. 1000–2700 m elev. in the Andes and Cauca River valley. In contrast, Marin et al. (2018) and Vigle et al. (2020) found it to be nested inside the montane clade. It is unclear if this contradiction is due to differences in character evidence (e.g. inclusion of phenomic evidence by Grant et al. 2017), taxon sampling (e.g. inclusion of additional *Leucostethus* species by Marin et al. 2018 and Vigle et al. 2020), or analytical methods (DNA sequence alignment and optimality criterion). Nevertheless, there seems little reason to doubt that *L.siapida* sp. nov. is sister to *L.bilsa*, which was recently described from ca. 340 km SW in the Reserva Biológica Bilsa at 420–515 m elev., northwestern Ecuador (Vigle et al. 2020).

Vigle et al. (2020) estimated a divergence time of approximately 3 Ma for *Leucostethusbilsa* and *L.siapida* sp. nov. Given that Gorgona has only been isolated from the mainland for at most 18–20 ka, and probably considerably less (Fleming et al. 1998; Montealegre-Z et al. 2010), isolation on the island appears not to have been causally related to the speciation event, suggesting that this is a relictual population of a formerly widespread species. Moreover, given the recency of the terrestrial connection between Gorgona and the mainland, we are optimistic that mainland populations will be discovered. Indeed, given its close resemblance to species of *Silverstoneia*, it is possible that specimens misidentified as *S.nubicola* already reside in natural history collections.

Among the species of *Leucostethus*, *L.siapida* sp. nov. most resembles *L.argyrogaster* and *L.fugax* from western Amazonia at 340–870 m elev. in Ecuador and Peru. In addition to lacking dark ventral coloration, these three species share orange flash marks (reported for *L.argyrogaster* by Morales and Schulte 1993; shown for *L.fugax* by Grant et al. 2017: fig. 33B), and *L.siapida* sp. nov. and *L.argyrogaster* further share the lack of conspicuous swelling of finger IV in adult males. Despite their resemblance, phylogenetic analyses consistently place the cis-Andean species as sister taxa (for which the orange flash coloration is an apparent synapomorphy) that are far removed from *L.siapida* sp. nov.

Scoring the occurrence of swelling on finger IV of adult males is unproblematic in species that exhibit conspicuous swelling (developmental variation and preservation artifacts notwithstanding), as do most species of *Leucostethus*; however, distinguishing between absence and weak swelling is notoriously difficult (Grant et al. 2017). For example, although Grant et al. (2017) scored finger IV as unswollen in *L.siapida* sp. nov., subsequent detailed examination of external morphology indicated that it is weakly swollen, and histological analysis revealed the presence of a specialized mucous gland found exclusively in the swollen finger IV (I.R.S. Cavalcanti and TG, unpublished data). As such, extreme caution is required when employing absence versus weak swelling to diagnose similar species. For this reason, we did not distinguish between the reported absence of swelling in *L.argyrogaster* and *L.bilsa* and the weak swelling in *L.siapida* sp. nov.

A curious characteristic shared by *Leucostethussiapida* sp. nov. and its sister species *L.bilsa* is the lateral variation in testis melanization. Although ontogenetic variation in testis pigmentation is common, individual variation among adults is rare, and, prior to *L.bilsa* (Vigle et al. 2020), unilateral melanization in dendrobatoids had only been documented for *Colostethuspanamansis* (Dunn, 1933) by Grant (2004). Golberg et al. (2020) found that testicular melanization and germ cell differentiation proceed in parallel in four anuran species, but the significance of intra-individual variation is unknown.

To our knowledge, the miniscule, white spots on the posteroventral surface of the thighs have not been reported previously, possibly because they are only evident in life, but they are widespread in Dendrobatoidea. Although an exhaustive review was beyond the scope of the current study, they also occur in additional species of *Leucostethus* (e.g. *L.bilsa*; Vigle et al. 2020: figs 2–5) and a least some species of the aromobatid genera *Allobates*, *Anomaloglossus*, *Aromobates*, *Mannophryne*, and *Rheobates* and the dendrobatid genera *Colostethus*, “*Colostethus*” *ruthveni*, *Epipedobates*, *Hyloxalus*, and *Silverstoneia*, and we are unaware of their occurrence in *Ectopoglossus*, *Paruwrobates*, *Phyllobates*, Dendrobatini, or potentially close relatives of Dendrobatoidea, including bufonids, hylodids, and *Thoropa*. Further study is required to determine if the presence of these spots is a synapomorphy of Dendrobatoidea with a limited number of informative losses, as available data suggest, or if their evolutionary history is more complicated.

In addition to describing the new taxon *Leucostethussiapida* sp. nov., we transferred *L.alacris*, *L.dysprosium*, and *L.yaguara* from *Colostethus* to *Leucostethus*. Vigle et al. (2020: 368) also considered the generic placement of *C.alacris* and *C.yaguara* but concluded that “placing them in the genus *Leucostethus* requires more evidence or data (e.g. molecular and/or morphological) from type series or topotypical samples.” Nevertheless, it should not be overlooked that the persistence of these species in *Colostethus* for the past 15 years was not due to empirical evidence, but rather (1) their placement in *Colostethus* prior to its partitioning into multiple genera by Grant et al. (2006, 2017) and Marin et al. (2018), and (2) their overall resemblance to the species now referred to *Leucostethus*. Although referral of these species to *Leucostethus* is not based on either quantitative phylogenetic analysis or clear synapomorphies and is, therefore, more predictive than explanatory, it is not entirely unsubstantiated by morphology. All three species share with other species of *Leucostethus* the presence of a complete (i.e. groin to eye), continuous, pale oblique lateral stripe (incomplete in some specimens of *L.bilsa*). In contrast, the oblique lateral stripe is either absent (*C.thorntoni*, *C.ucumari*, and “*C.*” *ruthveni*; for phylogenetic relationships of the last species, see Grant et al. 2017), continuous but incomplete (extending from groin midway along flank in *C.furviventris*, *C.imbricolus*, *C.inguinalis*, *C.latinasus*, *C.panamansis*, and *C.pratti*), or complete but formed by a series of spots (*C.agilis*, *C.lynchi*, and *C.mertensi*).

Despite our optimism that additional populations of *Leucostethussiapida* sp. nov. will be found on the mainland, based on current knowledge, *L.siapida* is endemic to Gorgona Island. Although Gorgona Island is protected as Parque Nacional Natural (PNN) Gorgona, the fact that the only known population is confined to an island of only ca. 13 km^2^ is sufficient to categorize this species as Vulnerable according to the IUCN Red List criteria (criterion D2; IUCN 2012). Nevertheless, we emphasize that this categorization should not prevent studies of this species from being undertaken, as many of the most basic aspects of its morphology (e.g. larval morphology, ontogenetic variation) and behavioral ecology (e.g. courtship and amplexus, oviposition site, parental care) are unknown, and studies detailing its fine-scale distribution and abundance, age-sex structure, and the frequency of morphological abnormalities, such as the testicular anomaly reported herein, will be crucial for managers of PNN Gorgona to monitor and protect the species. Ultimately, the occurrence of this endemic vertebrate adds to an already lengthy list of reasons to consider Gorgona to be a crucial locality for biodiversity conservation (Giraldo et al. 2014).

## Supplementary Material

XML Treatment for Leucostethus siapida

## Footnotes

Sia is translated as the Spanish flecha, which can refer to either ‘arrow’ or ‘dart’ but is usually translated as ‘arrow.’ As noted by Myers and Daly (1976: 180; see also Myers et al. 1978), this is mildly misleading in some contexts, given that modern English usage consistently distinguishes between arrows (used with bows) and darts.

## Supplementary materials

Supplementary material 1 Audio S1 Data type WAV file Explanation note Advertisement call of adult male holotype of *Leucostethussiapida* sp. nov. (CPZ-UV 7293, 22.7 mm SVL; 26.2°C ambient temperature). Audio extracted from Video S1. https://binary.pensoft.net/file/582358This dataset is made available under the Open Database License (http://opendatacommons.org/licenses/odbl/1.0/). The Open Database License (ODbL) is a license agreement intended to allow users to freely share, modify, and use this Dataset while maintaining this same freedom for others, provided that the original source and author(s) are credited.Author: Taran Grant

Supplementary material 2 Audio S2 Data type WAV file Explanation note Advertisement call 1 of adult male paratype of *Leucostethussiapida* sp. nov. (CPZ-UV 7295, 19.9 mm SVL; 26.2°C ambient temperature). https://binary.pensoft.net/file/582359This dataset is made available under the Open Database License (http://opendatacommons.org/licenses/odbl/1.0/). The Open Database License (ODbL) is a license agreement intended to allow users to freely share, modify, and use this Dataset while maintaining this same freedom for others, provided that the original source and author(s) are credited.Author: Taran Grant

Supplementary material 3 Audio S3 Data type WAV file Explanation note Advertisement call 2 of adult male paratype of *Leucostethussiapida* sp. nov. (CPZ-UV 7295, 19.9 mm SVL; 26.2°C ambient temperature). https://binary.pensoft.net/file/582360This dataset is made available under the Open Database License (http://opendatacommons.org/licenses/odbl/1.0/). The Open Database License (ODbL) is a license agreement intended to allow users to freely share, modify, and use this Dataset while maintaining this same freedom for others, provided that the original source and author(s) are credited.Author: Taran Grant

Supplementary material 4 Video S1 Data type MOV file Explanation note Adult male holotype of *Leucostethussiapida* sp. nov. vocalizing in situ (CPZ-UV 7293, 22.7 mm SVL). https://binary.pensoft.net/file/582361This dataset is made available under the Open Database License (http://opendatacommons.org/licenses/odbl/1.0/). The Open Database License (ODbL) is a license agreement intended to allow users to freely share, modify, and use this Dataset while maintaining this same freedom for others, provided that the original source and author(s) are credited.Author: Taran Grant
